# Simultaneous detection of metabolite concentration changes, water BOLD signal and pH changes during visual stimulation in the human brain at 9.4T

**DOI:** 10.1177/0271678X221075892

**Published:** 2022-01-21

**Authors:** Johanna Dorst, Tamas Borbath, Karl Landheer, Nikolai Avdievich, Anke Henning

**Affiliations:** 1 High-Field MR Center, Max Planck Institute for Biological Cybernetics, Tübingen, Germany; 2 IMPRS for Cognitive and Systems Neuroscience, 9188 University of Tübingen, University of Tübingen, Tübingen, Germany; 3 Faculty of Science, 9188 University of Tübingen, University of Tübingen, Tübingen, Germany; 4 Regeneron Pharmaceuticals, Tarrytown, New York, USA; 5 Advanced Imaging Research Center, UT Southwestern Medical Center, Dallas, TX, USA

**Keywords:** Functional magnetic resonance spectroscopy, MC-semiLASER, ultrahigh magnetic field, visual stimulation

## Abstract

This study presents a method to directly link metabolite concentration changes and BOLD response in the human brain during visual stimulation by measuring the water and metabolite signals simultaneously. Therefore, the metabolite-cycling (MC) non-water suppressed semiLASER localization technique was optimized for functional ^1^H MRS in the human brain at 9.4 T. Data of 13 volunteers were acquired during a 26:40 min visual stimulation block-design paradigm. Activation-induced BOLD signal was observed in the MC water signal as well as in the NAA-CH_3_ and tCr-CH_3_ singlets. During stimulation, glutamate concentration increased 2.3 ± 2.0% to a new steady-state, while a continuous increase over the whole stimulation period could be observed in lactate with a mean increase of 35.6 ± 23.1%. These increases of Lac and Glu during brain activation confirm previous findings reported in literature. A positive correlation of the MC water BOLD signal with glutamate and lactate concentration changes was found. In addition, a pH decrease calculated from a change in the ratio of PCr to Cr was observed during brain activation, particularly at the onset of the stimulation.

## Introduction

Blood-oxygen-level-dependent (BOLD) functional magnetic resonance imaging (fMRI) is the most widely used method to study brain function.^1^ Since the measured signal is based on a mismatch of blood flow and oxygen metabolism during neural activation resulting in hyperoxygenation and changed magnetic susceptibility and, therefore, changed proton signal intensity, it does not provide quantitative information on neuronal activation.^2,3^ Functional magnetic resonance spectroscopy (fMRS) is used as a complementary tool to study metabolite concentration changes during brain activation. Early proton (^1^H) fMRS studies report lactate (Lac) changes in the human brain due to visual stimulation measured using J-editing methods.^4,5^ Also, more recent studies use J-edited and long TE methods to look specifically at lactate, γ-aminobutyric acid (GABA), and glutamate and glutamine (Glx) alterations under visual stimulation.^6–8^ While editing and long TE sequences intrinsically remove overlapping signals, therefore enabling precise assessment of changes in Lac, GABA, and Glx, short TE non-edited measurements are advantageous because of high SNR and the simultaneous detection of a wide range of brain metabolites. Several short TE non-edited ^1^H fMRS studies have previously been performed in the human brain under visual stimulation at an ultra-high field (UHF) strength of 7 T.^8–17^ They consistently report increases of lactate (Lac) and glutamate (Glu) during stimulation.^8–17^ Some of them also report concomitant decreases in aspartate (Asp) and glucose (Glc) and interpret these metabolite changes as increasing oxidative energy metabolism during neuronal activation.^9,10,12,15,18^

To strengthen the link between hemodynamic and neurochemical responses and to interpret observed metabolite alterations, several studies correlate metabolite concentration changes with the BOLD signal.^12,13,15,16,19^ However, most of them do not collect fMRI and fMRS data simultaneously. Typically, shorter stimulation blocks in the fMRI than in the fMRS measurements^12,15,16^ are employed, making a direct comparison of BOLD signal and metabolite concentration changes difficult.

Besides Lac and Glu concentration changes, animal ^1^H fMRS studies conducted at UHFs between 9.4 T and 14.7 T report a decrease of the phosphocreatine (PCr) concentration and an increase in the creatine (Cr) concentration during brain activation.^20–23^ The observed downregulation of PCr during stimulation is consistent with some phosphorus (^31^P) fMRS studies in the human brain,^24–27^ while others did not observe this downregulation.^28–31^ ^31^P fMRS studies not only report contradictory results on metabolite concentration changes but also for pH estimates. While some studies report that intracellular pH did not change due to human brain activation,^28,29,32,33^ others report a rise in pH^34,^^35^ or a decrease.^36,37^ These contradictory results might be a result of the limited spatial and temporal resolution of ^31^P MRS that makes it challenging to capture possible local dynamic fluctuations. ^1^H MRS offers a higher temporal and spatial resolution than ^31^P MRS. Besides, due to the improved spectral resolution at UHFs, PCr and Cr can be accurately quantified with ^1^H MRS. As demonstrated by Watanabe et al.,^38^ pH could be estimated based on the creatine phosphokinase equilibrium using the ratio of PCr/Cr concentrations. So far, no pH alterations measured with ^1^H fMRS in vivo were reported.

This study aims at simultaneous measurement of water and metabolite signals to directly link the hemodynamic and the neurochemical responses upon brain activation. Thereby, former limitations of non-simultaneous BOLD and metabolite signal measurements are overcome. For these simultaneous measurements, the non-water suppressed metabolite-cycling (MC) ^1^H MRS technique^39^ was employed for functional spectroscopy in the human brain for the first time. The chosen MC-semiLASER sequence together with the partial volume coil setup optimized for ^1^H fMRS in the human visual cortex and the increased spectral resolution and sensitivity at an ultra-high field strength of 9.4 T allowed robust quantification of functional changes in metabolite concentrations on a single volunteer basis. Next to Lac and Glu, this study design also enabled the observation of metabolite concentration changes in the PCr buffer system from separately fitted PCr and Cr metabolite concentrations. These PCr and Cr metabolite concentrations might additionally facilitate the calculation of pH alterations during visual stimulation of the human brain.

## Material and methods

### Hardware

All experiments were performed on a 9.4 T whole-body MRI scanner (Siemens, Erlangen, Germany) using a home-built 8-element half-volume ^1^H coil with 4 transceive (TxRx) overlapping loops and 4 receive only (Rx only) loops, positioned perpendicularly to the plane of TxRx loops. The coil was specially designed and optimized for fMRS in the human visual cortex.^40^ To optimize transmit RF field distribution in the visual cortex, we used only 3 out of 4 TxRx loops, which were driven with a phase difference of 90° between each. RF power was applied using an unbalanced three-way Wilkinson power splitter (Supporting Information Figure S1a). As a result, an average maximum B_1_^+^ of 63 µT ranging from 57 µT to 68 µT could be achieved in the voxel selected for spectroscopy measurements. A holder mounting with a mirror placed in front of the volunteers’ eyes was affixed to the coil, such that the volunteer could view the visual stimulus, projected onto a screen in the back of the magnet bore. The visual angle was 7.6° in height and 10.1° in width.

### Participants

In total, data from 13 healthy volunteers (7 male; 6 female; age: 28 ± 2.5 years) were acquired. All experiments were in accordance with local research ethics policies, the Declaration of Helsinki in its current version and DIN EN ISO 14 155 and were approved by the Institutional Review Board of the University of Tübingen. Written informed consent was given by all volunteers before the examination. The total measurement duration was 75 minutes per volunteer, and the study was well tolerated by all of them. For data analysis, data of three volunteers were discarded; two due to minor lipid contamination, and one since the volunteer could not correctly see the turning fixation cross in the stimulus presentation due to uncorrected astigmatism.

### Stimulation paradigm

**Figure 1.**
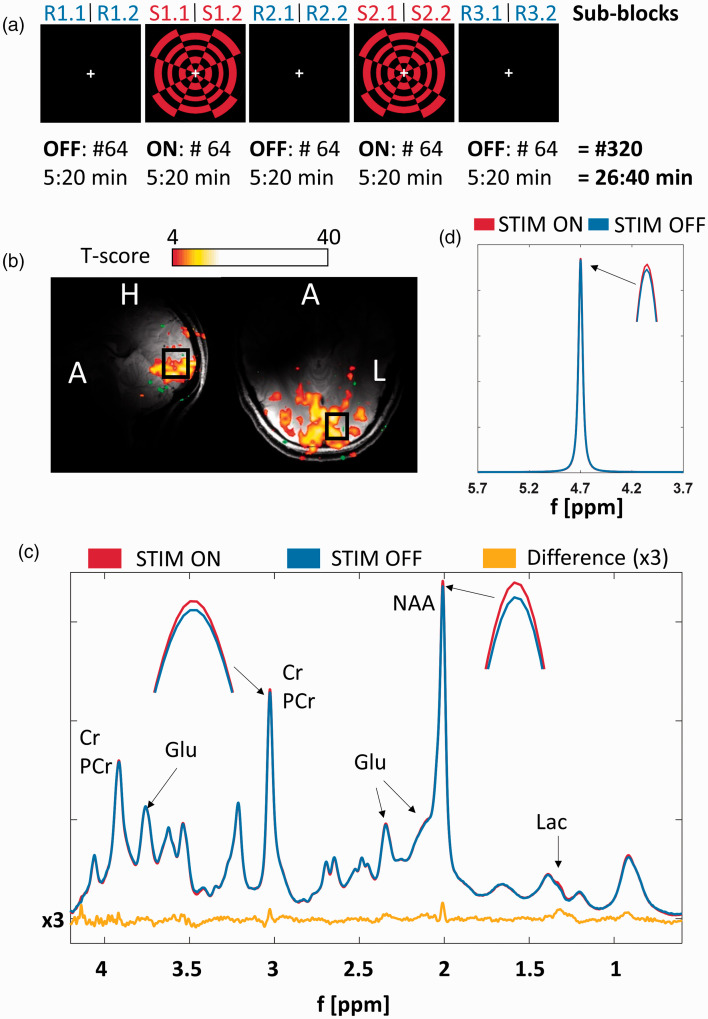
(a) Schematic diagram of the fMRS visual stimulation paradigm using a red-black checkerboard pattern. The sub-blocks are labeled as indicated above depiction of the visual paradigm (32 averages per sub-block, 2:40 min acquisition time). The numbers below indicate the acquired number of averages per block and the corresponding acquisition time. (b) FLASH images in sagittal and transversal directions overlaid by the fMRI t-score maps and the fMRS voxel position (15 × 18 × 20 mm^3^). (c) Representative MR spectra summed over all volunteers acquired during second halves of STIM (red, S1.2&S2.2) and REST (blue, R2.2&R3.2) periods (640 averages for each spectrum) and the difference spectrum, which was increased by a factor of three for better visualization (yellow). Small peak height and linewidth changes of tCr-CH_3_ and NAA-CH_3_ indicate the BOLD effect. (d) MC water spectra from one volunteer also acquired during second halves of STIM (red, S1.2&S2.2) and REST (blue, R2.2&R3.2) periods (64 averages for each spectrum). Small peak height and linewidth changes indicate the BOLD effect.

For strong and robust activation of the visual cortex, a visual stimulation paradigm similar to the one used in Mangia et al.^9^ was designed. The stimulus consisted of a radial red-black checkerboard flickering at 10 Hz (STIM). A dark screen was presented during rest periods (REST). A white fixation cross in the center of vision was used as a fixation point (Figure 1(a)). This cross changed its orientation randomly during the whole measurement. To track the volunteers’ attention, they were asked to press a button whenever the cross changed its orientation.

### MRS data acquisition

The anatomy of the occipital cortex was visualized by high-resolution images from 2D FLASH scans (field-of-view: 192 × 192 mm^2^, in-plane resolution: 0.6 × 0.6 mm^2^, slice thickness: 3 mm, 25 slices, TE/TR 9/378 ms, flip angle: 25°, acquisition time: 2:33 min) acquired in sagittal and transversal directions. A standard multislice Siemens-EPI sequence (field-of-view: 192 × 192 mm^2^, in-plane resolution: 3 × 3 mm^2^, slice thickness: 3 mm, 22 slices, TE/TR 20/2500 ms, flip angle: 65°, acquisition time: 2:35 min) was used to acquire BOLD-fMRI data. BOLD-fMRI data were acquired during a 2:30 min visual stimulation paradigm consisting of five blocks of alternating STIM (10 s) and REST (20 s) and directly analyzed during the measurement using the integrated Siemens software. These fMRI data guided the spectroscopy voxel placement (15 × 18 × 20 mm^3^) into an activation rich area of the left hemisphere of the occipital cortex (Figure 1(b)). After voxel placement, localized second-order shimming was performed using FASTESTMAP^41,42^ followed by voxel-based power calibration.^43^

The functional spectroscopy MR data were acquired using a metabolite-cycled (MC) semiLASER sequence (TE/TR 24/5000 ms).^44^ The slice-selective excitation was performed using a Hamming-windowed sinc excitation pulse with a duration of 1 ms, a flip angle of 90°, a time-bandwidth product of 8.75, and an excitation bandwidth of 8.75 kHz. For refocusing, two trapezoidal shaped adiabatic full passage (AFP) pulse pairs with a duration of 3.5 ms each were used.^45^ These pulses provide a bandwidth of 8 kHz at 9.4 T assuming an adiabatic threshold of 24 µT. As a result of the high excitation and inversion bandwidths of the pulses, the chemical shift displacement error (CSDE) was 5% per ppm in each voxel dimension. Since the transmit reference frequency of the scanner (ν_ref_) was set to 2.4 ppm, the CSDE in the upfield spectrum (CSDE of 5.5% for Lac at 1.31 ppm and 8.3% for mI at 4.05 ppm), as well as the outer volume lipid excitation, were minimized. The localization sequence was preceded by an asymmetric AFP pulse (composed of a sech and a tanh/tan pulse) used for metabolite cycling.^44,46^ The pulse duration was 22.4 ms, which provides a bandwidth of 1.7 kHz assuming an adiabatic threshold of 22 µT. The spoiler gradient and phase cycling schemes were optimized using DOTCOPS.^47,48^ Spoiler gradients were arranged as depicted in Supporting Information Figure S1b and described in Supporting Information Table S1 and had a spoiling momentum of 32 ms·mT/m. The spoiler gradients directly after the MC pulse had a spoiling momentum of only 7.5 ms · mT/m. For phase cycling, a 16-steps COG16(0,15,1,15,0,14;7) scheme was implemented,^47,49^ as described in Supporting Information Table S2. Phase cycling together with spoiler gradients removed all unwanted coherence pathways.

For metabolite spectra, a total of 320 averages were acquired during 26:40 min measurement time for the stimulus experiment and the control experiment each. The stimulus experiment included five blocks (REST-STIM-REST-STIM-REST) of 64 averages and a duration of 5:20 min each, while the control experiment included only REST blocks. To avoid any influence of the MC pulse on scaling the internal water reference signal, additional water reference spectra were acquired with semiLASER localization (TE/TR 24/5000 ms, 16 averages, ν_ref_ 4.7 ppm) without metabolite cycling. In addition, macromolecular spectra were acquired for each volunteer at rest with a double inversion recovery technique preceding the optimized MC-semiLASER localization (T_inv1_/T_inv2_ 2360 ms/625 ms, TE/TR 24/8000 ms, 32 averages, ν_ref_ 2.4 ppm).^50^ All spectroscopy measurements were carried out with an acquisition bandwidth of 8 kHz and 4096 complex sampling points.

### MRS data preprocessing

Raw data were processed with an in-house written MATLAB tool similar to Giapitzakis et al.^44^ The processing steps included: truncation of FIDs after 250 ms (512 ms were originally acquired) with subsequent zero-filling back to 4096 complex sampling points to increase SNR in the frequency domain for further processing; frequency and phase alignment in the time domain; rescaling and reconstruction of MC data;^39^ data averaging; zero-order phase correction and eddy current correction using the phase information of the MC water signal;^51^ combining signals from the eight receive loops using a singular-value decomposition algorithm based on the MC water signal;^52^ peak alignment of NAA to 2.008 ppm in the frequency domain; removal of residual water signal in the frequency domain using a Hankel singular value decomposition;^53^ final truncation of the FID to 150 ms (100 ms for macromolecular background spectra) with subsequent zero-filling back to 4096 complex sampling points.

### Assessment of the BOLD effect in water and metabolite spectra

For each volunteer, single-scan data were averaged in 10 sub-blocks (32 averages each) and afterwards scaled to the water reference and summed across all volunteers (10 volunteers × 32 averages = 320 averages per spectrum). To investigate the BOLD effect, peak heights and linewidths were evaluated for each spectrum (time resolution 2:40 min) by upsampling by a factor of 20 in post-processing and finding the maximum peak height as well as the full width at half maximum using an in-house written MATLAB function. For NAA-CH_3_ (acetyl moiety at 2.008 ppm) and total Cr (methyl group (CH_3_) of Cr and PCr at 3.028 ppm), peak heights and linewidths were deduced from LCModel fits that were imported in MATLAB. Importing the LCModel fits into MATLAB permitted the separation of the metabolite spectrum from the overlapping macromolecules and other metabolite resonances. Since Cr and PCr were fitted separately in LCModel, these peaks were combined to tCr in MATLAB before evaluation of peak heights and linewidths. For MC water, the BOLD effect was directly investigated from preprocessed spectra. To assess the statistical relationship between MC water, NAA-CH_3_ and tCr-CH_3_ peak heights and linewidths of the 10 time points from spectra summed across all volunteers, Spearman’s correlation analysis was applied calculating Spearman’s Rank Correlation Coefficient (R) and the significance level of the correlation (p-value).

### fMRS data analysis

To investigate the metabolite concentration changes during STIM and REST, single-scan data were averaged in three different ways: (1) single-scan data from the first halves of the two STIM blocks (S1.1 & S2.1; see Figure 1(a)) were summed resulting in a spectrum of 64 averages (2 × 32 averages) per volunteer; the same was done for the second and third REST blocks (R2.1 & R3.1); this is called ‘1st’ in the following. The first REST block was omitted from this analysis to have STIM and REST spectra with the same number of averages. (2) The same was done with the second halves of STIM (S1.2 & S2.2) and REST (R2.2 & R3.2) blocks each; this is called ‘2nd’ in the following. (3) Lastly, all single-scan data from STIM (S1.1, S1.2, S2.1, S2.2) and REST (R2.2, R2.2, R3.1, R3.2) were summed, resulting in two spectra of 128 averages each per volunteer (2 × 64 averages); this is called ‘all’ in the following.

Metabolites from all of these spectra were quantified using LCModel version 6.3-1 L^54^ (see Supporting Information Figure S2 for sample spectra with metabolites). LCModel analysis was performed over the spectral range from 0.6 ppm to 4.1 ppm with DKNTMN set to 0.25 to reduce the probability of under- or overestimation of metabolite concentrations due to a highly flexible spline baseline.^55^ A physically realistic metabolite basis set was simulated in MARSS^56^ via the quantum mechanical density matrix formalism. The simulation employed experimentally realistic shaped RF pulses, gradients, timings and a sufficient number of spatial points to accurately model the voxel sidebands. The following 17 metabolites were simulated: ascorbic acid (Asc), aspartate (Asp), total choline (tCho, glycerophosphocholine (GPC) + phosphocholine (PCho)), creatine (Cr), phosphocreatine (PCr), γ-aminobutyric acid (GABA), glucose (Glc), glutamate (Glu), glutamine (Gln), glutathione (GSH), lactate (Lac), myo-inositol (mI), N-acetylaspartate (NAA), N-acetylaspartylglutamate (NAAG), phosphoethanolamine (PE), scyllo-inositol (Scyllo), taurine (Tau). For measured macromolecule spectra, the visible residual methylene group resonance of tCr-CH_2_ (3.92 ppm) was fitted as a Voigt line using LCModel. The fitted residual tCr-CH_2_ resonance was then subtracted from the macromolecular spectra to obtain a pure macromolecule spectrum.^57,58^ The macromolecular spectra free of residual metabolite signals were then scaled to the water reference, summed over all volunteers and included into the fitting procedure as a macromolecule basis spectrum. The water reference spectra were used as internal scaling reference in LCModel, and metabolite concentrations are given in arbitrary units with respect to the water reference without correction for relaxation effects.

To check whether the metabolite concentration changes between STIM and REST are statistically significant, a non-parametric Wilcoxon signed-rank test (α = 0.05) was applied. To control for the Type 1 error rate (incorrect rejection of the null hypothesis) arising from multiple testing, the Benjamini-Hochberg False-Discovery-Rate correction (q = 0.05) was applied.

The metabolite concentration difference relative to the baseline concentration (second half of first REST block, R1.2) was visualized with an increased time resolution of 40 s (37 spectra with 32 averages each were fitted for each volunteer) using a moving average with a sliding offset of 8 scans for every volunteer. The mean and the standard deviation were calculated.

To examine the relationship of metabolite concentration changes and the water BOLD effect during the stimulus paradigm, single-subject data were averaged into 10 sub-blocks (2:40 min each). The sub-blocks were then scaled to the water reference and summed across all volunteers. These summed spectra were finally quantified in LCModel. Metabolite concentrations were correlated to the MC water peak height for the 10 time points using Spearman’s correlation, similarly to the metabolite BOLD effect assessments described above.

### pH estimation

Intracellular pH in the human brain was calculated from the creatine phosphokinase equilibrium $\mathrm{PCr}+\mathrm{ADP}+H^{+}↔Cr+\mathrm{ATP}$ according to

$$pH=-\log_{10}\left(\frac{\left[\mathrm{ATP}\right]\times \left[Cr\right]\times K^{\prime }}{\left[\mathrm{ADP}\right]\times \left[\mathrm{PCr}\right]}\right)$$

where $K^{\prime }=7.09\times 10^{-9}$ at 37°C is the apparent equilibrium constant, and a constant $\left[\mathrm{ATP}\right]/\left[\mathrm{ADP}\right]=11.47$ ratio taken from the literature^38,59^ was assumed. Cr and PCr concentrations were taken from spectra fitted with a time resolution of 40 s (see section ‘fMRS data analysis’). Mean pH changes during STIM and REST were calculated from the first halves of STIM and REST blocks (‘1st’), the second halves of STIM and REST blocks (‘2nd’) and for the whole STIM and REST blocks (‘all’) similarly to how metabolite concentration changes were evaluated (see section ‘fMRS data analysis’).

## Results

### BOLD effect in water and metabolites

**Figure 2.**
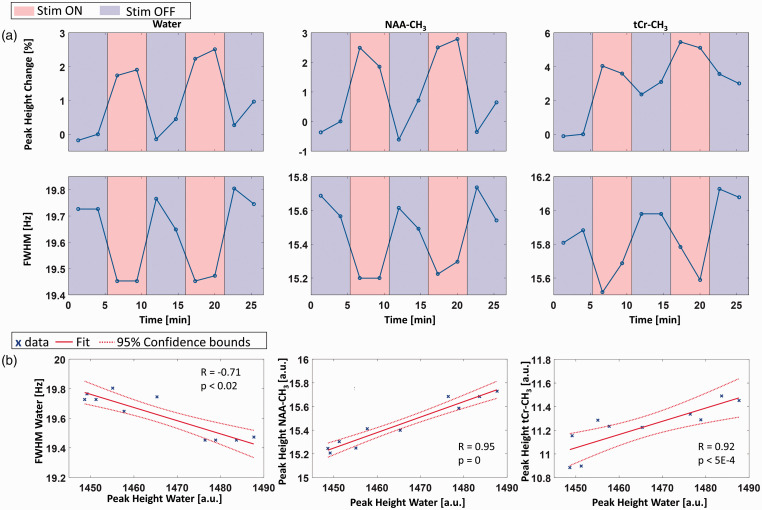
(a) Time course of peak heights and linewidths of MC water, NAA-CH_3_ and tCr-CH_3_ in functional experiments from spectra summed over all ten volunteers (320 averages per data point, 10 sub-blocks). Red colored backgrounds indicate the STIM periods, blue colored backgrounds the REST periods. (b) Scatter plots showing the correlation between the linewidth and peak height of MC water, and the correlations between the peak height of MC water and the peak height of NAA-CH_3_ and tCr-CH_3_ for the time points of the ten sub-blocks (blue crosses, 320 averages per data point). Solid red lines represent the linear fit, dotted red lines 95% confidence intervals. R and p values indicate the Spearman’s Rank Correlation Coefficient and the significance level of the correlation, respectively.

The brain areas activated by the visual stimulation paradigm were observable at the scanner console and used for MRS voxel planning. Figure 1(b) shows a representative fMRI t-score map of voxels that were significantly activated by visual stimulation from a representative volunteer. Supporting Information Figure S3 shows the t-score maps from all 10 volunteers. The black rectangle indicates the voxel dimensions (15 × 18 × 20 mm^3^) and position. The voxel was always placed in the activated region within the left hemisphere. The activation led to an increase in peak heights and decrease of linewidths for NAA-CH_3_ and tCr-CH_3_, as demonstrated in representative MR spectra summed over 10 volunteers acquired during STIM (red, S1.2 + S2.2) and REST (blue, R2.2 + R3.2) in Figure 1(c) (640 averages per spectrum). For better visualization, the difference spectrum (yellow) was increased by a factor of three. Peak height increase and linewidth decrease was also observed in the MC water signal (Figure 1(d)). Quantitative results of BOLD induced peak height and linewidth changes of these two metabolites as well as of MC water calculated from spectra summed over all ten volunteers are displayed in Figure 2. The peak height changes of the spectra summed across all volunteers caused by visual stimulation are similar between NAA-CH_3_, tCr-CH_3_ and the MC water (NAA-CH_3_: +2.3 ± 0.3%, tCr-CH_3_: +1.5 ± 0.6%, Water: +1.9 ± 0.2%). Consistent linewidth decreases [NAA-CH_3_: −0.37 ± 0.02 Hz (−2.4 ± 0.1%), tCr-CH_3_: −0.40 ± 0.03 Hz (−2.5 ± 0.2%), Water: −0.29 ± 0.04 Hz (−1.5 ± 0.2%)] were also observed along with the peak height increases. As expected, the linewidth changes of MC water of stimulus experiment data correlate with the respective MC water peak height changes (R = −0.71, p < 0.02, Figure 2(b)). The peak heights of NAA-CH_3_ and tCr-CH_3_ highly correlate with the MC water peak height (peak height NAA-CH_3_ – peak height water: R = 0.95, p = 0, peak height tCr-CH_3_ – peak height water: R = 0.92, p < 9E-4). Also, linewidth changes of NAA-CH_3_ and tCr-CH_3_ highly correlate with MC water peak height changes (linewidth NAA-CH_3_ – peak height water: R = −0.80, p < 5E-3, linewidth tCr-CH_3_ – peak height Water: R = −0.56, p < 0.09).

### Metabolite concentration changes during visual stimulation and their correlation to the BOLD effect

The high spectral quality obtained in this study at UHF (see Supporting Information Figure S4) in combination with an accurately simulated basis set that considers real RF pulse shapes and measurement parameters enabled the quantification of 15 individual metabolites and one combined metabolite (tCho) with estimated standard deviations of the LCModel fit %SD < 10% for most metabolites. Only metabolites with low concentrations and/or highly J-coupled spin systems, such as Asc, GABA, Lac, PE and Scyllo had a higher %SD < 20%. Glucose could not reliably be fitted in all spectra and was thus not included in further analysis. Metabolite baseline concentrations scaled to the water reference and their related estimated standard deviations are presented in Table 1. The data were quantified from fMRS spectra acquired during the second half of the first REST period (R1.2, 32 averages per spectrum, 2:40 min acquisition time). The first half of the first REST block (R1.1) was omitted from the analysis since the acquired signal at the beginning of each spectroscopy experiment might not yet have reached its steady-state amplitude. The respective steady-state magnetization is influenced by T_1_ relaxation effects in dependence of sequence parameters including flip angle and repetition time T_R_. In addition, Table 1 presents relative metabolite concentration changes between different time periods of REST and STIM conditions analyzed for the functional as well as the control experiment. Significant increases in metabolite concentrations (q < 0.05) during STIM were found for Glu and Lac for all analyzed time periods due to visual stimulation. The mean concentration changes of Glu and Lac based on water scaled LCModel baseline concentrations are nearly the same (Glu: 0.43; Lac: 0.46). While Glu increase was constant over the stimulation blocks, Lac increase was higher in the second halves of the stimulation blocks (2nd) than at the onset of stimulation (1st). When only considering the first halves of STIM and REST blocks (‘1st’), also a significant increase in Cr and decrease in PCr could be observed. No statistically significant changes were found in the control experiment.

**Table 1.** LCModel group analysis (10 volunteers) of metabolite baseline concentrations (second half of first REST, 32 averages per spectrum) and their changes during different time periods of STIM and REST (1st: first 32 single-scan spectra of STIM/REST summed; 2nd: second 32 single-scan spectra of STIM/REST summed; all: all 64 single-scan spectra of STIM/REST summed) for the functional as well as the control experiment.

	Functional experiment					Control experiment				
	Baseline Conc.	% SD	Concentration difference (STIM-REST) [%]			Baseline Conc.		Concentration difference (STIM-REST) [%]		
			1st	2nd	all		% SD	1st	2nd	all
Asc	2.8 ± 1.3	14.6 ± 17.2	−9.0 ± 20.2	−5.3 ± 24.5	−0.2 ± 25.6	3.1 ± 1.1	10.6 ± 6.6	−2.7 ± 54.6	28.5 ± 81.3	9.7 ± 59.0
Asp	7.0 ± 1.6	7.5 ± 2.5	15.5 ± 24.3	−4.8 ± 31.9	2.5 ± 21.0	8.8 ± 3.2	6.4 ± 2.0	5.4 ± 29.3	7.8 ± 34.0	2.7 ± 15.1
Cr	10.7 ± 1.3	3.3 ± 0.5	15.9 ± 22.3*	2.5 ± 20.1	6.1 ± 14.7	11.9 ± 2.2	3.3 ± 0.5	4.9 ± 22.1	5.1 ± 25.4	4.5 ± 20.6
PCr	10.3 ± 2.2	3.7 ± 0.9	−13.2 ± 15.1*	2.1 ± 28.8	−5.3 ± 16.4	10.8 ± 1.6	3.8 ± 0.9	3.5 ± 35.6	4.2 ± 31.3	1.9 ± 23.9
GABA	3.5 ± 1.6	8.9 ± 3.0	14.4 ± 38.4	−0.8 ± 14.3	3.7 ± 10.1	2.8 ± 1.0	11.9 ± 6.4	7.7 ± 41.9	−16.4 ± 41.4	3.7 ± 43.7
Glc	−	−	−	−	−	−	−	−	−	−
Gln	11.0 ± 1.1	2.4 ± 0.5	2.9 ± 4.6	0.3 ± 4.7	1.4 ± 3.1	10.5 ± 1.2	2.7 ± 0.5	−1.6 ± 4.3	0.9 ± 8.3	−0.6 ± 3.2
Glu	21.7 ± 1.5	1.3 ± 0.5	2.0 ± 2.1*	2.6 ± 2.6*	2.3 ± 2.0*	21.7 ± 1.2	1.4 ± 0.5	0.1 ± 3.4	0.3 ± 3.3	0.1 ± 2.5
GSH	2.4 ± 0.5	6.2 ± 1.5	2.1 ± 13.4	3.5 ± 20.9	1.2 ± 10.9	2.7 ± 0.4	5.6 ± 1.0	8.7 ± 12.1	9.7 ± 21.3	7.9 ± 9.7
Lac	1.3 ± 0.2	9.5 ± 1.8	26.3 ± 34.4*	52.9 ± 40.3*	35.6 ± 23.1*	1.2 ± 0.5	13.4 ± 7.7	−5.5 ± 10.0	3.4 ± 35.5	−3.2 ± 15.0
mI	17.7 ± 1.4	1.3 ± 0.5	2.8 ± 4.0	3.9 ± 5.0	4.3 ± 3.7	18.0 ± 2.2	1.5 ± 0.5	−0.1 ± 5.4	1.6 ± 4.0	0.6 ± 1.9
NAA	29.2 ± 1.6	1.0 ± 0	0.5 ± 1.7	0.03 ± 1.5	−0.03 ± 1.4	29.6 ± 1.3	1.0 ± 0.0	−0.3 ± 1.6	0.7 ± 2.3	−0.1 ± 1.6
NAAG	4.6 ± 0.5	3.3 ± 0.5	2.5 ± 6.4	−1.2 ± 5.3	1.4 ± 5.1	4.4 ± 0.4	3.7 ± 0.5	−0.4 ± 6.9	−2.4 ± 4.5	−0.7 ± 3.9
tCh	2.7 ± 0.4	2.3 ± 0.5	−1.4 ± 11.8	−5.0 ± 12.9	−3.7 ± 7.5	2.6 ± 0.6	2.6 ± 0.7	0.9 ± 8.4	1.3 ± 9.2	0.6 ± 6.9
PE	3.7 ± 2.5	16.0 ± 24.3	4.9 ± 38.5	11.4 ± 69.3	14.3 ± 85.0	4.4 ± 2.5	8.1 ± 4.5	9.0 ± 56.4	49.4 ± 131.4	13.2 ± 40.9
Scyllo	0.4 ± 24.3	15.1 ± 8.4	4.9 ± 47.7	15.8 ± 56.4	14.8 ± 44.5	0.5 ± 0.1	12.6 ± 3.7	30.6 ± 107.7	19.0 ± 32.9	17.6 ± 48.5
Tau	3.1 ± 0.8	6.4 ± 1.8	6.1 ± 19.5	14.0 ± 37.4	9.2 ± 24.9	3.1 ± 0.8	6.7 ± 1.6	0.8 ± 11.8	4.9 ± 16.5	3.5 ± 10.3

Statistically significant concentration differences (STIM−REST) assessed with a Wilcoxon signed-rank test (α = 0.05) combined with a Benjamini-Hochberg correction (q = 0.05) are marked with an asterisk.

**Figure 3.**
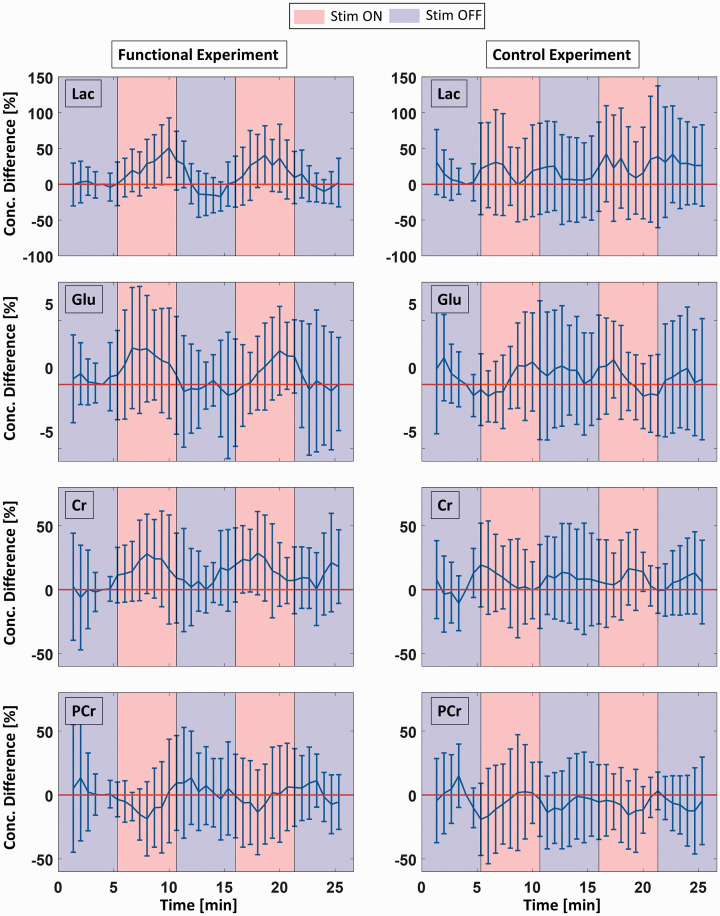
Mean sliding average time courses of Lac, Glu, Cr and PCr concentration differences relative to a baseline concentration (second half of first REST block, R1.2) with a time resolution of 40 s for the functional (left column) as well as the control experiment (right column). Error bars represent standard deviations of the mean over all ten volunteers. Red colored backgrounds indicate STIM periods, blue colored backgrounds REST periods.

**Figure 4.**
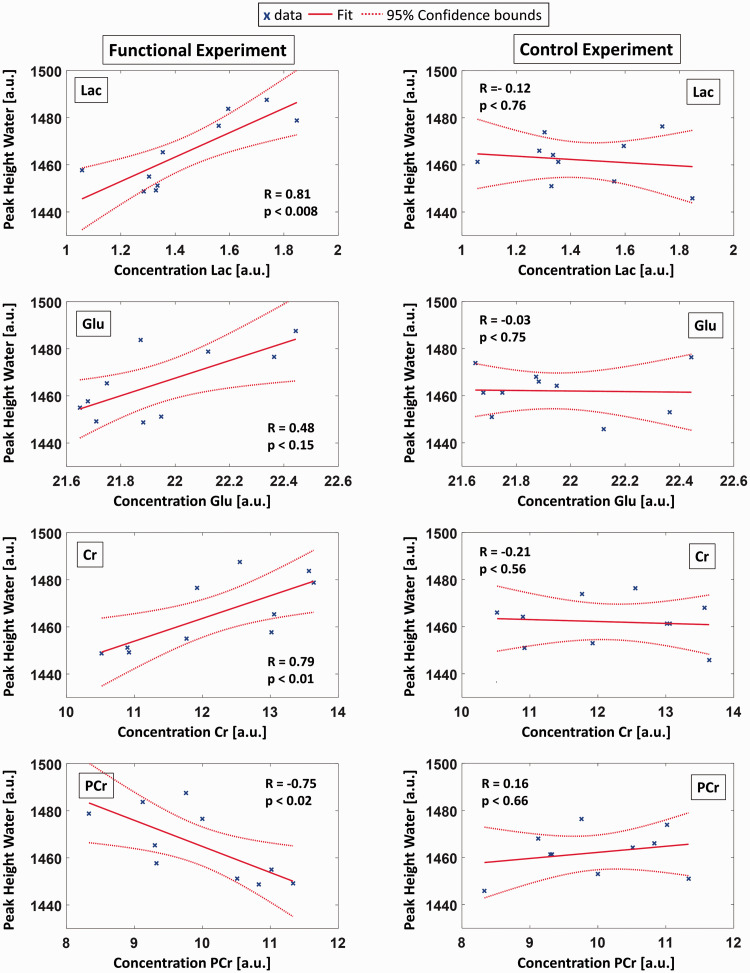
Scatter plots showing the correlation between the metabolite concentrations of Lac, Glu, Cr and PCr and the peak height of MC water for the time points of the ten sub-blocks (blue crosses, 320 averages per data point). Results from the functional experiment are shown in the left column and from the control experiment in the right column. Solid red lines represent the linear fit, dotted red lines 95% confidence intervals. R and p values indicate the Spearman’s Rank Correlation Coefficient and the significance level of the correlation, respectively.

To illustrate the effects of stimulation on Lac, Glu, Cr and PCr, mean sliding average time courses of their concentration changes relative to the baseline concentration and their standard deviations (n = 10) are shown in Figure 3 with a time resolution of 40 s. As demonstrated in Figure 4, Lac, Glu, Cr and PCr concentration changes correlate with the simultaneously acquired MC water peak heigh changes in the functional experiment (peak height water – concentration Lac: R = 0.81, p < 8E-3, peak height water – concentration Glu: R = 0.48, p < 0.15, peak height water – concentration Cr: R = 0.79, p < 1E-2, peak height water – concentration PCr: R = −0.75, p < 2E-2). No correlation could be detected in control experiments.

### pH changes

**Figure 5.**
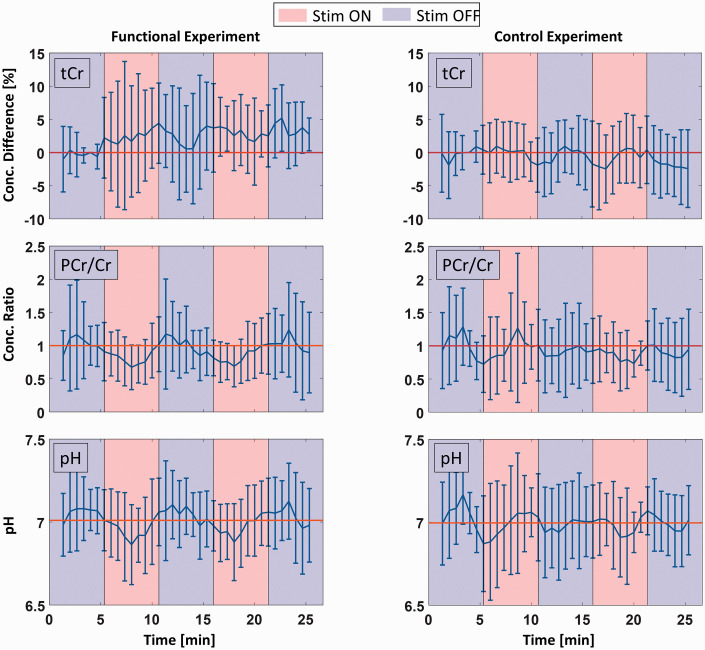
Mean sliding average time courses of total creatine (Cr + PCr) concentration differences relative to a baseline concentration (second half of first REST block, R1.2), the concentration ratio of PCr/Cr, and pH. The time resolution was 40 s, and the functional experiment results are shown in the left column while the control experiment results are shown in the right column. Error bars represent standard deviations of the mean over all ten volunteers. Red colored backgrounds indicate STIM periods, blue colored backgrounds REST periods. For the total creatine curve of the functional experiment, data of only nine volunteers were summed since one dataset revealed a baseline concentration drift.

Since statistical analysis revealed changes in Cr and PCr concentrations due to visual stimulation at the beginning of the block diagram, their behavior was further analyzed. Sliding average time courses shown in Figure 5 reveal drops of the phosphocreatine-to-creatine ratio (PCr/Cr) due to visual stimulation. As a reference, the time course of tCr (Cr + PCr) is shown for the functional experiment, which does not reveal any concentration change due to visual stimulation. There are also no changes in the PCr/Cr ratio and tCr concentration in the control experiments. Under the assumption of a constant ATP/ADP ratio, mean calculated pH during the whole acquisition time was the same for the functional and the control experiment with 7.01 ± 0.07 and 7.00 ± 0.06, respectively (see Table 2). The sliding average time course of pH follows the one of PCr/Cr following the pH formula given in ‘Material and Methods – pH estimation’. While there is no significant pH change during the control experiment, pH significantly drops at stimulation’s onset (ΔpH = 0.14, p = 0.002).

**Table 2.** Mean pH during the whole measurement of 26:40 min, and mean pH difference between different time periods of STIM and REST (1st: mean pH of first halves of STIM/REST; 2nd: mean pH of second halves of STIM/REST; all: mean pH of whole STIM/REST) for the functional as well as the control experiment.

	Functional experiment	Control experiment
Mean pH	7.01 ± 0.07	7.00 ± 0.06
Mean pH STIM, 1st	6.94 ± 0.04	6.97 ± 0.05
Mean pH REST, 1st	7.08 ± 0.03	6.98 ± 0.04
ΔpH, 1st	0.14*	0.01
Mean pH STIM, 2nd	6.99 ± 0.05	7.01 ± 0.06
Mean pH REST, 2nd	7.00 ± 0.03	6.99 ± 0.03
ΔpH, 2nd	0.01	-0.02
Mean pH STIM, all	6.95 ± 0.05	6.98 ± 0.06
Mean pH REST, all	7.04 ± 0.05	6.96 ± 0.03
ΔpH, all	0.09	0.02

Statistically significant concentration differences (STIM−REST) assessed with a Wilcoxon signed-rank test (α = 0.05) are marked with an asterisk.

## Discussion

For fMRS studies, the acquisition of artifact-free spectra is mandatory to see the small concentration changes induced by the stimulation. Therefore, the MC-semiLASER sequence was carefully optimized for human brain studies at 9.4 T regarding RF pulses, sequence timing, gradient spoiling and phase cycling. The use of this highly optimized MC-semiLASER sequence in combination with the ultra-high field strength of 9.4 T and a half-volume coil with optimized sensitivity in the occipital lobe allowed the acquisition of high-quality and highly reproducible MR spectra from a small voxel in the visual cortex. Additionally, the combination of semiLASER localization with the metabolite-cycling technique facilitated the simultaneous detection of metabolite concentrations and the water signal.

To acquire high-quality spectra with high resolution, not only the hardware setup and the localization sequence, but also shimming is an important step in data acquisition. The spectral resolution is dependent on B_0_ field homogeneity and field strength.^60^ The linewidth in spectroscopy is determined by T_2_ and macroscopic and microscopic susceptibility.^61^ T_2_, as well as microscopic susceptibility effects, cannot be eliminated by B_0_ shimming and contribute substantially to the spectral linewidth. The macroscopic susceptibility was strongly reduced in this study using state-of-the-art FASTESTMAP^41^ B_0_ shimming that was recommended for single-voxel shimming in a recent consensus paper.^42^ Nassirpour et al.^62^ compared different optimization algorithms for localized in vivo B_0_ shimming at 9.4 T. She showed that higher-order shim fields than second-order do not further improve B_0_ shimming results for single-voxel applications. Even though she optimized a constrained regularized algorithm for higher-order localized B_0_ shimming using an insert shim, we decided to use FASTESTMAP based on her results, and to measure without the insert shim to keep the measurement setup simple, robust and fully compatible with the fMRI stimulation setup. The spectral resolution obtained is comparable to former studies conducted at 9.4 T.^44,63^

In most previous studies, the BOLD signal was collected before fMRS data^12,15–17,64^ or in different scan sessions^65^ using significantly shorter stimulus block lengths in fMRI measurements than in fMRS measurements. Since the neurochemical response varies depending on the stimulation block length and block repetitions,^12,64^ and physiological, cognitive, and hardware related changes may occur between different scans, simultaneous acquisition of BOLD and fMRS data are critical to evaluate the link between hemodynamics and neurochemistry. So far, measurements combining fMRI-MRS or water-suppressed and water-unsuppressed measurements in the same TR provide the closest link between hemodynamics and neurochemistry.^6,13,19,66^ Since a 3D EPI sequence is used in the BOLD fMRI-MRS combining studies, activation maps with a high spatial resolution could be obtained. Interleaving EPI and MRS may affect the M_0_ relaxation process, may increase the temporal resolution of the MRS measurement due to SAR limitations, and potential eddy current effects from the EPI readout may influence MRS data quality.^13,19,67^ Nevertheless, the BOLD effect associated with a line narrowing of the water signal due to brain activation is examined in fMRI measurements in almost all of the mentioned studies.^2,3,8,10–12,15^ One additional study examines the BOLD effect from water unsuppressed spectra, albeit measured before the metabolite fMRS examination using a different stimulation paradigm.^12^ Only one recently published study demonstrates the power of concurrent fMRI-fMRS measurements interleaving unsuppressed water acqusitions with water-suppressed J-difference editing acqusitions.^6^ In this study, we demonstrated the BOLD induced line narrowing behavior due to brain activation in the MC water signal acquired simultaneously with the metabolite signals from the same voxel. The BOLD effect on water is easier to compute than on metabolites since metabolite peaks are overlapped by macromolecules and other metabolite resonances, which makes linewidths and peak heights determination challenging. In addition, the simultaneous acquisition of the water BOLD and metabolite signals enables a direct temporal comparison of water BOLD and neurochemical response. However, since the combination of metabolite cycling and phase cycling used in this study requires 32 averages for full 3D voxel localization, the temporal resolution of the water BOLD signal is limited to 2:40 min. The line narrowing of the MC water signal correlates with the MC water peak height during the stimulation periods. Linewidth decrease and peak height increase during the stimulation phase have also been observed for the singlets in the simultaneously measured metabolite spectra. The signal peak height of tCr-CH_3_ and NAA-CH_3_ increased by ∼2% during the stimulation periods, similar to the peak height increase in MC water. This metabolite peak height increase is in good agreement with previously reported peak height increases of 3%^11^ and 2%^9,65^ at 7 T. Since a high correlation of linewidths and peak height changes of metabolites with the MC water signal changes could be shown, it can be assumed that the main cause of linewidth changes of the metabolites tCr-CH_3_ and NAA-CH_3_ is associated with the BOLD effect.^68^

The high quality and reproducibility of the spectra allowed reliable detection of the time course of 15 metabolites for each single volunteer. We opted not to correct the small line-narrowing due to the BOLD effect in STIM spectra before LCModel quantification, as done in previous studies.^9–13,15,17,19,64^ The linewidth is a fit parameter in LCModel and is automatically corrected for in the analysis. The linewidth correction before LCModel fitting did not affect quantification results in previous 7 T studies beyond 1%.^12,64^ However, applying exponential multiplication to FIDs to correct line broadening effects introduces additional possible error sources and artificially reduces the spectral resolution. Since the linewidth changes of the singlets NAA-CH_3_ and tCr-CH_3_ between STIM and REST, taken for line broadening corrections in literature, are slightly different, they would need to be averaged. Also, the noise level of spectra change upon exponential multiplication and this would need to be corrected as well. However, the profound alterations of the magnetic properties due to brain activation may also modify the lineshape. To precisely correct the distorted lineshape before fitting is challenging. To correct one effect (linewidth), but ignore the other one (lineshape) may mask real effects.

The group analysis results show significant increases in Lac and Glu concentrations during visual stimulation, which is in good agreement with previous studies conducted at 7 T.^8–12,15,17^ In the literature, significant changes have also been reported for various other metabolites such as Asp, Glc, GABA, GSH and Gly, which were not observed in our study.^9–12,14–16^ This might be a result of different experimental setups, such as differences in sequences, voxel positioning and size, stimulus characteristics, or other data processing. The Glu time course observed in this study showed an increase of ∼2.3% within the first minute reaching a new steady-state level and remaining unchanged till the end of the stimulation period. This is consistent with the Glu time courses observed in previous studies for similar stimulation periods.^8–13,15–17^ The mean Lac increase of 35.6 ± 23.1% over the whole stimulus period detected in this study is slightly higher than the literature values of 7 to 30% increase.^8–12,14,15,17^ Whereas the studies by Mangia et al.^9,65^ and Schaller et al.^11^ show an increase in Lac to a new steady-state within the first minute of activation, Lin et al.^10^ observed a transient Lac increase with a subsequent return toward baseline despite the ongoing stimulus. In contrast to these studies, a continuous increase in Lac during 5:20 min stimulation periods was observed in the present study as can be seen in Figure 3 and from Table 1. In contrast to previous studies that only analyzed MRS spectra from the second half of the stimulus blocks,^9,12,65^ the metabolite concentration changes of the first halves of the stimulus blocks were also analyzed in this study. This approach is justified by the fact that the hemodynamic response delay is in the range of only a few seconds while the stimulus block length is several minutes. The investigation of first and second halves of the stimulus block allowed for a more detailed investigation of temporal changes of metabolite concentrations throughout the visual stimulation phase. The respective finding of a continuous increase in Lac is in good agreement with a previously published study of Fernandes et al.,^8^ who observed a gradual increase during the first 7-8 minutes of stimulation.

Positive correlations between the metabolite concentration changes of Lac and Glu and the MC water peak height due to the BOLD effect were observed. These correlations demonstrate the link between brain energy metabolism via Lac and Glu detection and the respective hemodynamic response assessed via the BOLD effect. These findings are in line with previous fMRI/fMRS studies^12,13,15,16^ that did not yield simultaneous assessment of water BOLD effect and metabolite concentration changes.

Even though ^1^H fMRS is feasible for functional investigation of the human brain, the interpretation of observed metabolite changes is limited by the low spatial and temporal resolution, the missing information about changes in metabolic fluxes and technical challenges for reliable quantification of metabolite concentration changes with low effect size.^18^ Therefore, the results obtained in this study may be interpreted by different biological mechanisms. Increases in Lac and Glu reflect increased brain energy demands during visual stimulation periods.^12,18^ A related reduction in Glc, which was observed in several previous studies,^10–12,15^ could not be determined in our study. Previously, it was suggested that the Lac and Glu changes represent an increased flux into the oxidative pathway^9,69,70^ after its adjustment towards a higher steady state. Additionally, an increase in Lac could also reflect an intensification of anaerobic glycolysis^8,14,71^ or could arise as a product of astrocytic glycolysis.^18^ The increase of Glu was interpreted as a sign of increased TCA cycle due to its dynamic equilibrium with the TCA cycle rate intermediate alpha-ketoglutarate (α-KG).^72,73^ Another possibility for increased Glu concentrations is de novo synthetization of Glu which can be transferred to the neurons for use as neurotransmitter.^70^ Further possible explanations for the observed increase in Glu concentrations might be the concept of an increased Glu/Gln cycling rate during neural stimulation, which mediates Glu transportation to neurons,^70,71^ or an increased flux through the malate-aspartate shuttle.^9,10,12,15,72^

Besides Lac and Glu concentration increases, and a peak height increase and linewidth narrowing in tCr-CH_3_ due to visual stimulation, significant changes in PCr and Cr concentrations could be observed. Although the methyl protons of these two metabolites overlap with each other (Cr: 3.027 ppm; PCr: 3.029 ppm^74^) in our spectra, the high SNR and spectral resolution along with a very narrow transition band of the MC pulse allows separate fitting of Cr and PCr based on their methylene peaks (Cr: 3.913 ppm; PCr: 3.930 ppm^74^). The decrease in the PCr/Cr ratio, which is more pronounced at the onset of stimulation, is consistent with results obtained in ^1^H fMRS rodent studies^20–23^ and a PCr signal decrease observed in some ^31^P fMRS studies in humans.^24–27^ PCr serves as a source of high energy phosphates. To maintain a constant ATP concentration during the onset of brain activation, the creatine phosphokinase equilibrium is shifted towards the formation of ATP at the expense of PCr.^20,75,76^ PCr serves as a buffer and the respective creatine kinase reaction is the fastest process under physiologic conditions to generate ATP.^77^ The PCr buffer system bridges the time until oxidative phosphorylation in the TCA cycle starts to produce more ATP to fulfill the energy needs of the brain during activation.^78^ Therefore, the pronounced changes in PCr/Cr ratio were observed in the beginning of stimulation blocks. Increased creatine kinase exchange flux due to visual stimulation has also been shown in ^31^P magnetization transfer studies in humans at different field strengths.^27,31,33,79^ Since our results of PCr and Cr concentration time courses under visual stimulation confirm previous results,^20–27,31,33,79^ we used these concentration ratios to additionally calculate pH. The mean pH value of ∼7 calculated is consistent with intracellular pH values reported in ^31^P brain studies at 7 T and 9.4 T.^80–82^ Under the assumption of a constant ATP/ADP ratio, we also calculated pH time courses. The mean pH decrease of 0.09 due to visual brain activation calculated in this study is slightly higher than the pH changes calculated from the PCr/Cr ratios obtained in other ^1^H fMRS studies for visual stimulation in tree shrews (ΔpH = 0.04)^22^ and for forepaw stimulation in rats (ΔpH = 0.08)^23^. An intracellular pH decrease is in accordance with a shift in the creatine phosphokinase equilibrium to form ATP at the expense of PCr accompanied by proton uptake.^38,76^ The pH values and time courses calculated in this study confirm previous results.^22,23,36,37,80–82^ However, several ^31^P fMRS studies report increasing pH values upon brain activation.^27,34,35^ This might be a result of the measurements at different field strengths, acquisition parameters and stimuli, as well as differences in volunteers' age. However, our method to calculate pH does not take the ATPase reaction into account, but assumes a constant ATP/ADP ratio. The ATP/ADP ratio cannot be measured with the used experimental setup. To precisely calculate temporal pH changes taking the creatine kinase reaction as well as the ATPase reaction fully into account, a combination of ^1^H and ^31^P fMRS is needed. Alternatively, the simultaneous acquirement of upfield and downfield ^1^H spectra could be used to validate pH values calculated from the PCr/Cr ratio with pH values calculated from homocarnosine.^83^ A limitation of the pH estimation using the PCr/Cr ratio is the spectral overlap and respective correlation between the concentration estimates of these two metabolites as reported in the LCModel .print files (−0.90 ± 0.01 in REST and STIM conditions).

## Conclusions

MC-semiLASER was successfully applied for fMRS to simultaneously study metabolite concentration changes and the MC water BOLD signal in the activated human brain. It confirms previously detected metabolite changes in Lac and Glu, and their correlation to the MC water BOLD signal affirms that the stimulus-induced concentration changes are related to increased energy metabolism as a result of increased neuronal activity. Due to the high quality of the spectra measured at 9.4 T, separation of Cr and PCr becomes feasible, thereby enabling non-invasive pH measurements. pH decrease during brain activation is hypothesized to correspond to a shift in the creatine kinase equilibrium towards the formation of ATP.

## Supplemental Material

sj-pdf-1-jcb-10.1177_0271678X221075892 - Supplemental material for Simultaneous detection of metabolite concentration changes, water BOLD signal and pH changes during visual stimulation in the human brain at 9.4T Click here for additional data file. Supplemental material, sj-pdf-1-jcb-10.1177_0271678X221075892 for Simultaneous detection of metabolite concentration changes, water BOLD signal and pH changes during visual stimulation in the human brain at 9.4T by Johanna Dorst, Tamas Borbath, Karl Landheer, Nikolai Avdievich and Anke Henning in Journal of Cerebral Blood Flow & Metabolism
