# Supplementary material for: Simultaneous detection of metabolite concentration changes, water BOLD signal and pH changes during visual stimulation in the human brain at 9.4T
Source: J Cereb Blood Flow Metab. 2022 Jan 21;42(6):1104–19. doi: 10.1177/0271678X221075892 (PMC9121534; doi:10.1177/0271678X221075892)
Supplement: sj-pdf-1-jcb-10.1177_0271678X221075892 - Supplemental material for Simultaneous detection of metabolite concentration changes, water BOLD signal and pH changes during visual stimulation in the human brain at 9.4T [file sj-pdf-1-jcb-10.1177_0271678X221075892.pdf]

Supporting Information

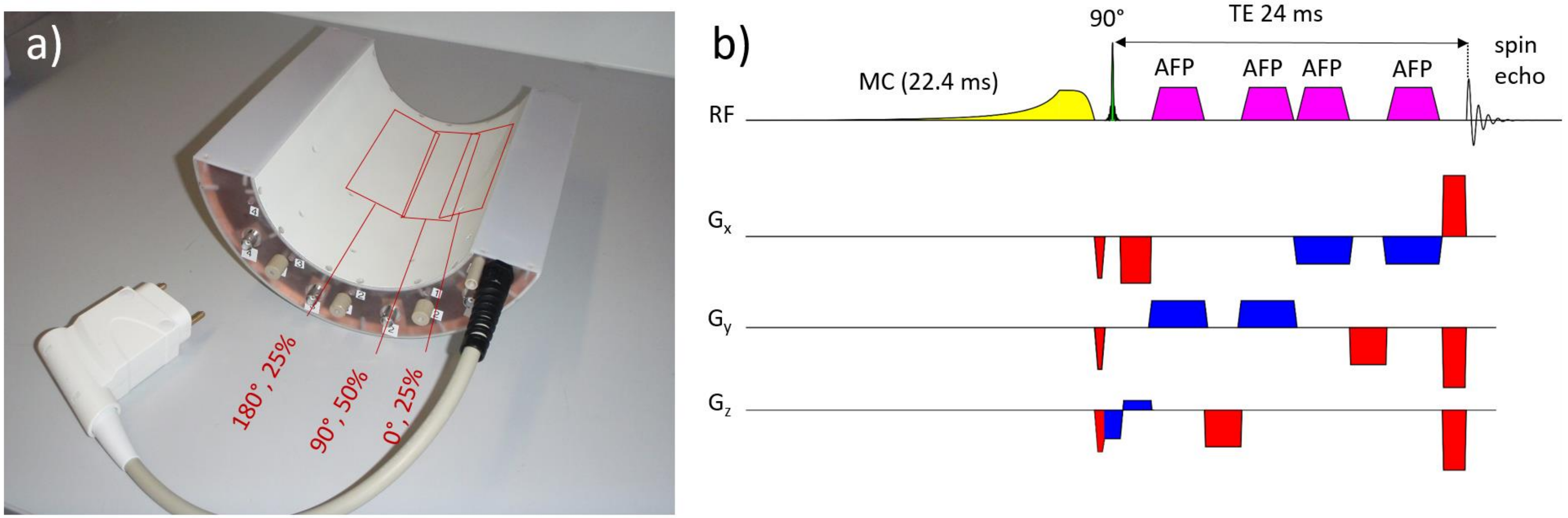

Supporting Information Figure S1: (a) Photo of the half-volume ^{1}H coil with the three transceiver (TxRx) loops, their phase increment and the power distribution illustrated[1]. (b) Schematic of MC-semiLASER (TE 24 ms) with an asymmetric adiabatic full passage MC pulse (22.4 ms pulse duration, yellow) preceding a Hamming-windowed sinc excitation pulse (1 ms pulse duration, 8.75 kHz bandwidth, green) and two trapezoidal-shaped adiabatic full passage refocusing pulse pairs (3.5 ms pulse duration, 8 kHz bandwidth, magenta). Spoiler gradient timing and direction during the sequence are indicated, spoiler gradients at the end of the sequence are not shown. Spoiler gradients are marked in red, localization gradients in blue.

Crusher Position	Spoiling momentum x-axis [ms·mT/m]	Spoiling momentum y-axis [ms·mT/m]	Spoiling momentum z-axis [ms·mT/m]
**After MC Pulse**	7.5	7.5	7.5
**After sinc Pulse**	32.0	0	0
**After 1st AFP**	0	0	32.0
**After 2nd AFP**	0	0	0
**After 3rd AFP**	0	32.0	0
**After 4th AFP**	-32.0	0	0

Supporting Information Table S1: Spoiling momenta of the crusher gradients carried out in the MC-semiLASER sequence[2].

Crusher No	MC Pulse	Sinc Pulse	1st AFP	2nd AFP	3rd AFP	4th AFP	ADC
**1**	0	0	0	0	0	0	0
**2**	0	15/8	1/8	15/8	0	7/4	7/8
**3**	0	7/4	1/4	7/4	0	3/2	7/4
**4**	0	13/8	3/8	13/8	0	5/4	5/8
**5**	0	3/2	1/2	3/2	0	1	3/2
**6**	0	11/8	5/8	11/8	0	3/4	3/8
**7**	0	5/4	3/4	5/4	0	1/2	5/4
**8**	0	9/8	7/8	9/8	0	1/4	1/8
**9**	0	1	1	1	0	0	1
**10**	0	7/8	9/8	7/8	0	7/4	15/8
**11**	0	3/4	5/4	3/4	0	3/2	3/4
**12**	0	5/8	11/8	5/8	0	5/4	13/8
**13**	0	1/2	3/2	1/2	0	1	1/2
**14**	0	3/8	13/8	3/8	0	3/4	11/8
**15**	0	1/4	7/4	1/4	0	1/2	1/4
**16**	0	1/8	15/8	1/8	0	1/4	9/8

Supporting Information Table S2: COG16(0,15,1,15,0,14;7) phase cycling scheme in the MC-semiLASER sequence[3]. Columns represent the different pulses in the localization sequence in chronological order. The last column represents the receiver phase. The units of the phases are π radians.

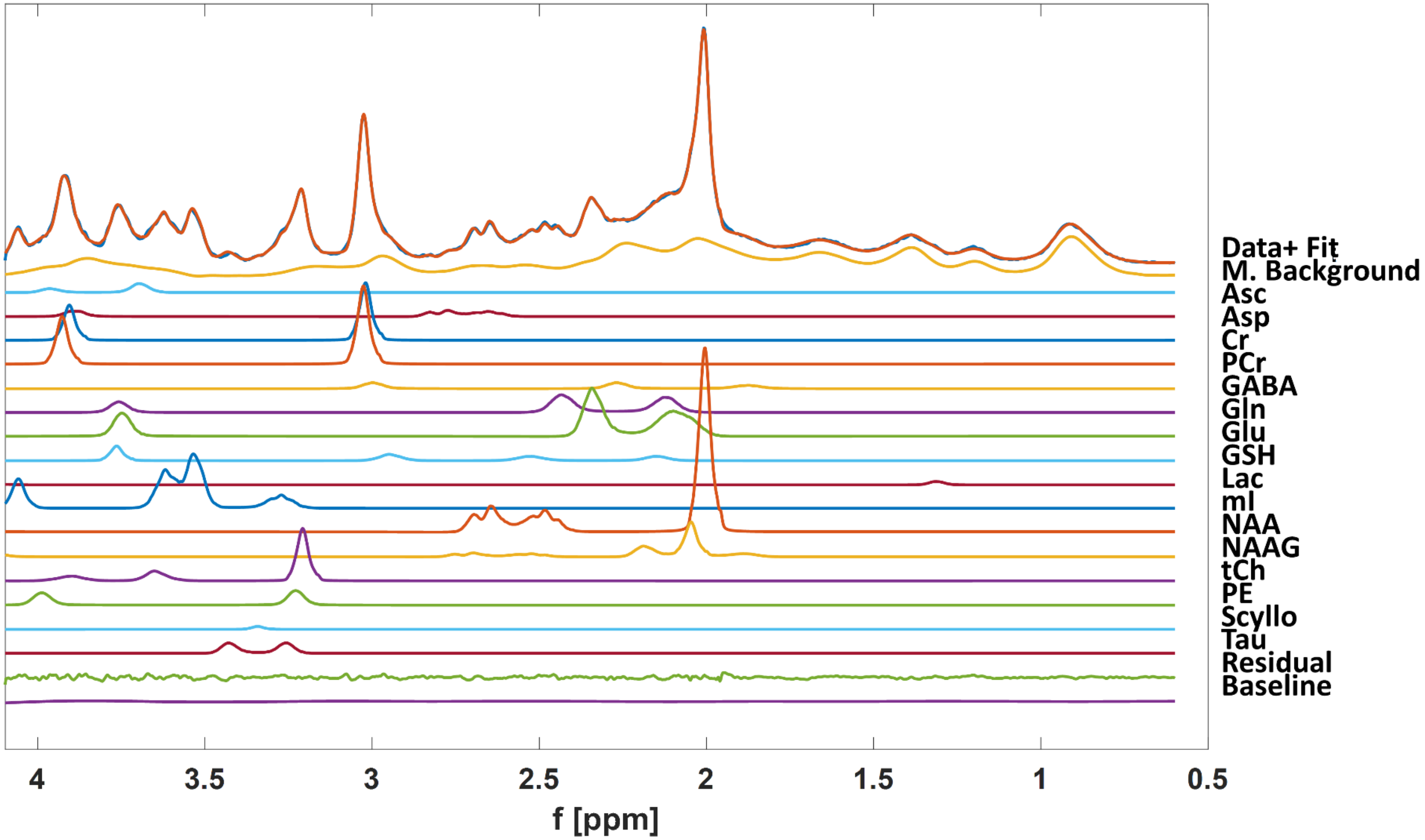

Supporting Information Figure S2: LCModel quantification sample spectrum (64 averages) of one volunteer with the fitted metabolites. Abbreviations: MMB: macromolecular baseline; Asc, ascorbic acid; Asp, aspartate; Cr, creatine; PCr, phosphocreatine; GABA, γ-aminobutyric acid; Gln, glutamine; Glu, glutamate; GSH, glutathione; Lac, lactate; mI, myo-inositol; NAA, N-acetyl aspartate; NAAG, N-acetyl aspartyl glutamate; tCh, combined phosphocholine, glycerophosphocholine; PE, phosphoethanolamine; Scyllo, scyllo-inositol; Tau, taurine.

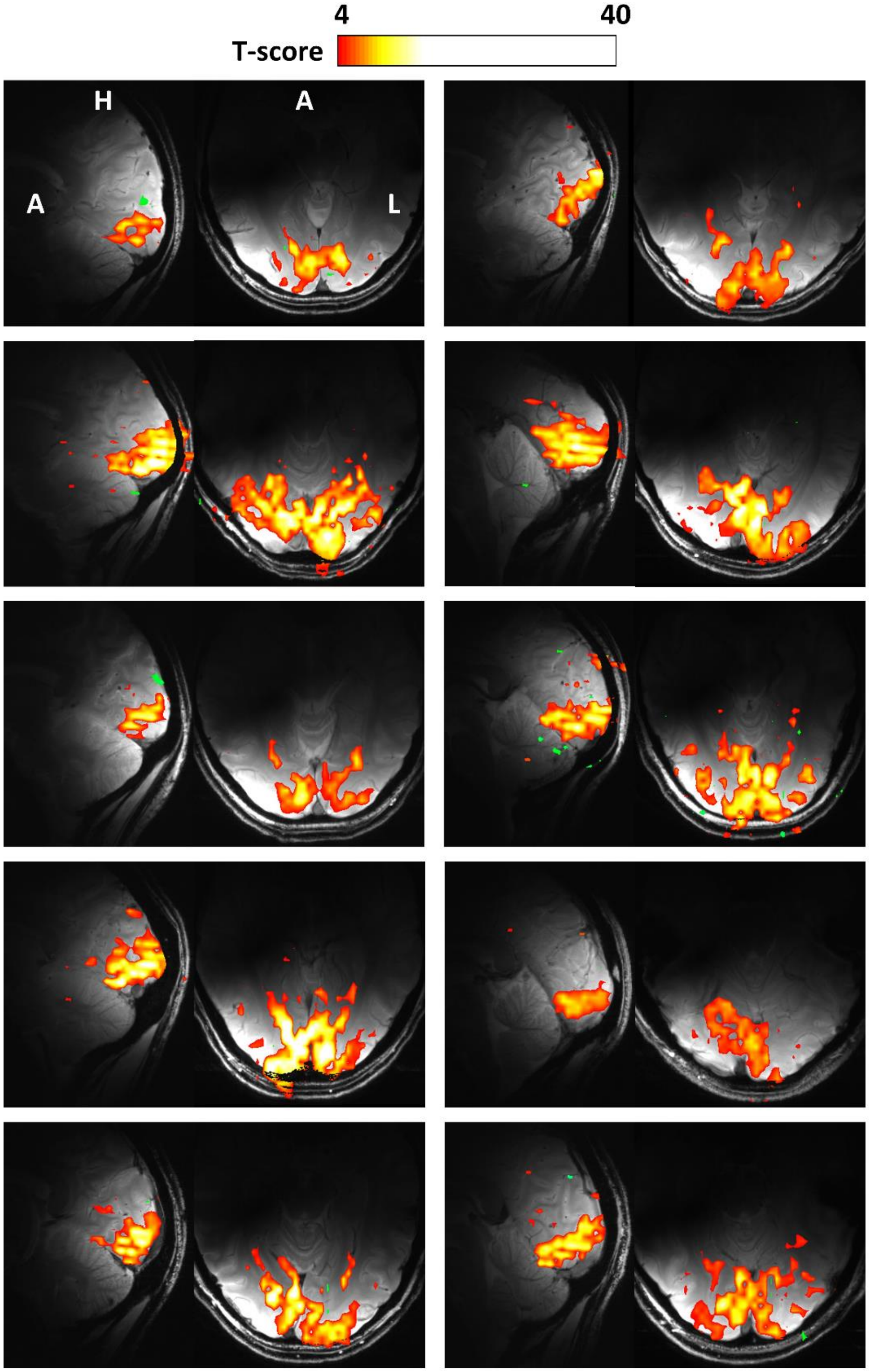

Supporting Information Figure S3: FLASH images in sagittal and transversal directions from all 10 volunteers overlaid by the fMRI t-score maps.

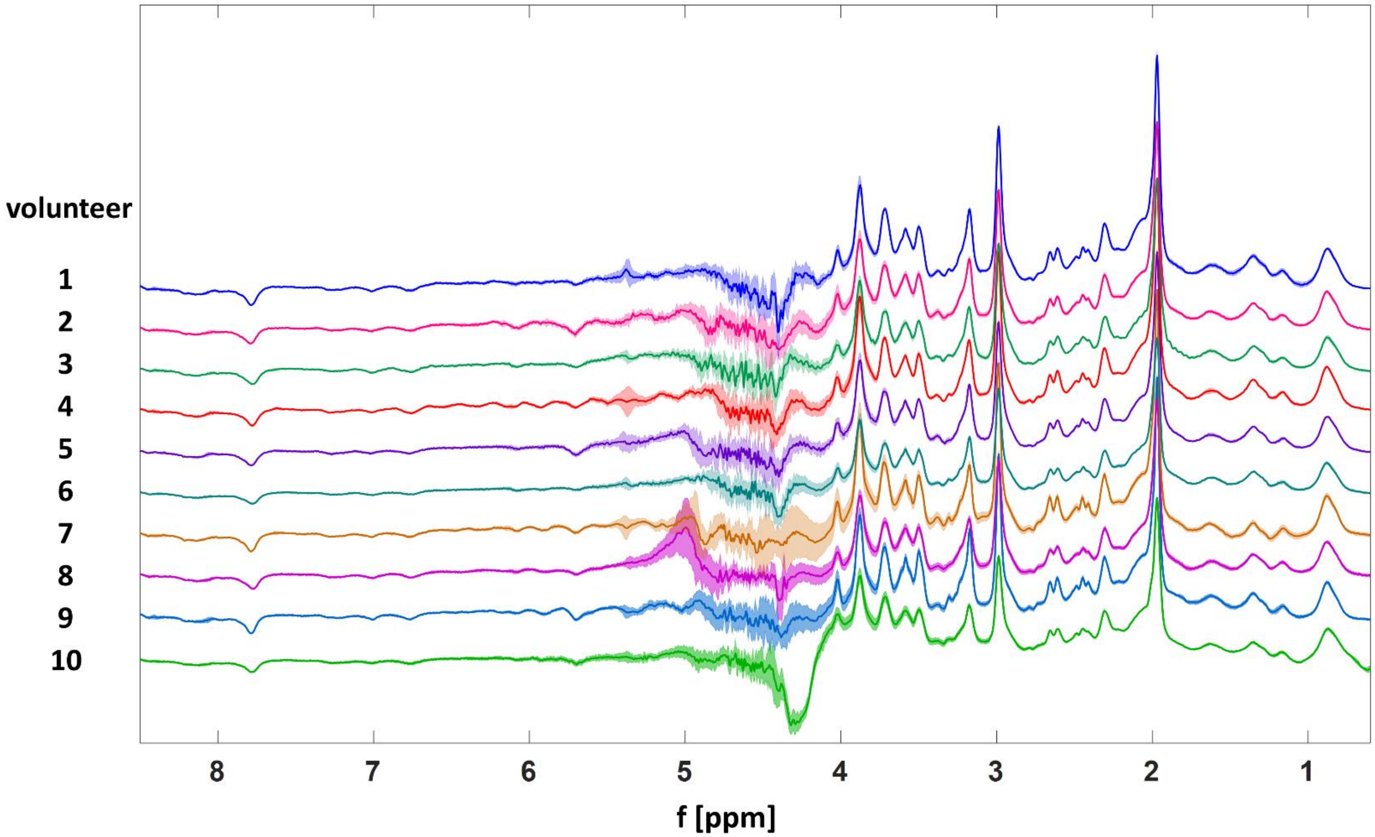

Supporting Information Figure S4: Spectra of all ten volunteers acquired during the control experiment demonstrating the reproducibility. The acquired data of all volunteers were divided into ten subblocks (32 averages, 2:40 acquisition time). Solid lines show the mean of the ten subblocks per volunteer, and the transparent areas indicate standard deviations across subblocks. Mind that the downfield spectrum is shown inverted due to the MC sequence.

References:

1. Pfrommer A, Avdievich NI, Henning A. Four Channel Transceiver Array for Functional Magnetic Resonance Spectroscopy in the Human Visual Cortex at 9.4 T. In: *Proceedings of the Joint Annual Meeting ISMRM-ESMRMB, 2014, Milano, Italy*. 2014, p. 1305.

2. Landheer K, Juchem C. Dephasing optimization through coherence order pathway selection (DOTCOPS) for improved crusher schemes in MR spectroscopy. *Magn Reson Med* 2019; 81: 2209–2222.

3. Landheer K, Juchem C. Simultaneous optimization of crusher and phase cycling schemes for magnetic resonance spectroscopy – an extension of DOTCOPS. *Magn Reson Med* 2020; 83: 391–402.
